# Association between continuity of care and treatment outcomes in psychiatric patients in Germany: a prospective cohort study

**DOI:** 10.1186/s12888-023-04545-x

**Published:** 2023-01-19

**Authors:** Werner de Cruppé, Michaela Assheuer, Max Geraedts, Karl Beine

**Affiliations:** 1 Institute for Health Services Research and Clinical Epidemiology Philipps-Universität Marburg, Karl-Von-Frisch-Strasse 4, 35043 Marburg, Germany; 2 School of Medicine, Faculty of Health, Institute for Health Systems Research, Herdecke University, Alfred-Herrhausen-Strasse 50, 58448 WittenWitten, Germany

**Keywords:** Psychiatric patient treatment, Continuity of care, Symptom severity, Social functioning, Quality of life, Psychiatric health care services research, Cohort study, Germany

## Abstract

**Background:**

Continuity of care is considered an important treatment aspect of psychiatric disorders, as it often involves long-lasting or recurrent episodes with psychosocial treatment aspects. We investigated in two psychiatric hospitals in Germany whether the positive effects of relational continuity of care on symptom severity, social functioning, and quality of life, which have been demonstrated in different countries, can also be achieved in German psychiatric care.

**Methods:**

Prospective cohort study with a 20-months observation period comparing 158 patients with higher and 165 Patients with lower degree of continuity of care of two psychiatric hospitals. Patients were surveyed at three points in time (10 and 20 months after baseline) using validated questionnaires (CGI Clinical Global Impression rating scales, GAF Global Assessment of Functioning scale, EQ-VAS Euro Quality of Life) and patient clinical record data. Statistical analyses with analyses of variance with repeated measurements of 162 patients for the association between the patient- (EQ-VAS) or observer-rated (CGI, GAF) outcome measures and continuity of care as between-subject factor controlling for age, sex, migration background, main psychiatric diagnosis group, duration of disease, and hospital as independent variables.

**Results:**

Higher continuity of care reduced significantly the symptom severity with a medium effect size (*p* 0.036, eta 0.064) and increased significantly social functioning with a medium effect size (*p* 0.023, eta 0.076) and quality of life but not significantly and with only a small effect size (*p* 0.092, eta 0.022). The analyses of variance suggest a time-independent effect of continuity of care. The duration of psychiatric disease, a migration background, and the hospital affected the outcome measures independent of continuity of care.

**Conclusion:**

Our results support continuity of care as a favorable clinical aspect in psychiatric patient treatment and encourage mental health care services to consider health service delivery structures that increase continuity of care in the psychiatric patient treatment course. In psychiatric health care services research patients’ motives as well as methodological reasons for non-participation remain considerable potential sources for bias.

**Trial registration:**

This prospective cohort study was not registered as a clinical intervention study because no intervention was part of the study, neither on the patient level nor the system level.

## Background

Continuity of care is a multidimensional construct [1] and indispensable in high-quality healthcare. There is agreement that continuity of care is particularly important to mental healthcare because of its often long-lasting and recurrent treatment episodes and important psychosocial treatment aspects. However, in the literature there is disagreement about a precise definition of continuity of care [2–4] but core dimensions of global concepts of continuity in mental healthcare could be identified [5]. In a multidisciplinary review, Haggerty et al. resume three types of continuity of care: informational continuity, management continuity and relational continuity. In this study, continuity of care means relational continuity: “an ongoing therapeutic relationship between a patient and one or more providers” and “the relationship is typically established with a team rather than a single provider” [6]. Continuity of care is generally considered to have a positive influence on the treatment of psychiatric patients [7–15] yet we find ongoing comparisons of personal continuity versus specialization of care as favorable care concepts for mental health services especially in European countries [16–18]. And a recent systematic review on personal continuity of the Swedish Agency for Health Technology Assessment points out the advantages on patients with severe mental illness but states their still low certainty of evidence [19]. Studies reporting better outcomes in continuously treated patients show an improvement in the level of social functioning measured using the GAF (Global Assessment of Functioning) [8, 9, 20, 21], quality of life [11, 12, 21–23] and symptom severity [11, 12, 22, 23]. Besides effectiveness, the efficiency of psychiatric treatment can be increased. Van der Lee et al. conclude in their study that continuity of care is related to less medical costs [24].

The few German studies investigating the topic continuity of care find similar results as the international literature, but only include selected diagnosis groups or patients of individual health insurance funds in the study population [25]. Karow et al. conclude for patients with psychosis treated continuously by a specialized ACT-team (Assertive Community Treatment) in comparison to patients in routine care a higher improvement in psychotic symptoms, severity of illness, social functioning, quality of life and treatment satisfaction [26]. In addition, they performed a cost-effectiveness analysis and point out a better efficiency of this ACT-program than for routine care [27].

The German health care system is characterized by sector boundaries (inpatient, day patient, outpatient) with different remuneration systems making it difficult or even impossible to implement continuity of care in everyday care. Since 2012, psychiatric hospitals in Germany can conclude a contract with health insurance funds on model projects with the key feature of an individual annual global treatment budget. This budget is not based on the costs and treatment episodes per setting or sector, but on the total number of persons treated per year independent of the sector where hospitals decide to treat them. A recent mixed-method process evaluation study finds three core mechanisms of these model projects. They provide hospitals more flexible and need-adapted treatment options for their patients, favor continuity of care and help patients to maintain everyday life [28]. A meta-analysis of 13 controlled cohort studies comparing claims data of more than twenty-six thousand patients from 13 hospitals with a contract for model projects all over Germany with matched patients of 13 control psychiatric hospitals without a model project shows significantly shorter inpatient care in hospital-new patients of model hospitals and a reduced duration of sick leave in those hospitals with longer preexisting contracts of model projects [29, 30]. Included were patients regardless of psychiatric diagnostic group and sickness fund, but claims data do not provide detailed psychiatric clinical data, nor data on relational continuity of care. An evaluation of a model project in northern Germany indicates on the one hand no reduction in the total mental health care costs for such programs but advantages regarding the cost-effectiveness [31], and on the other hand an improved level of social functioning for patients [32, 33] but without focusing on continuity of care.

To understand if a model project might enhance the relational continuity of care, we conducted a cohort study with an observation period of 20 months comparing patients from a model hospital with patients from a hospital without model project. We could show that the relational continuity of care in patients of a model hospital was significantly higher than for patients of a hospital without model project. This counted for the inpatient treatment at the time of recruitment as well as across all settings during the observation period. Details of these analyses of our cohort study are published elsewhere [34].

In a subsequent analysis of our cohort study, we wanted to study if continuity of care affects clinical outcomes. Therefore, we hereinafter present the results of our analyses on the association of relational continuity of care with clinical outcomes such as symptom severity, level of functioning, and quality of life.

## Methods

### Study design

Data analyzed are from a prospective cohort study comparing patients of a psychiatric hospital with a contract of a model project (model hospital) with patients of a psychiatric hospital without this type of contract as a control (control hospital). The observation time covers 20 months with primary data collection at recruitment (t0), at first follow-up after 10 months (t1), and at second follow-up after 20 months (t2) between May 2016 and October 2018.

### Study setting

Both psychiatric hospitals are located in North Rhine-Westphalia, a Federal Land in the central western part of Germany. They are equivalent in respect of structural features (inpatient care, day care, outpatient service, number of beds, staff, and patients treated), the spectrum of psychiatric diagnostic and treatment procedures provided, and both are responsible for the compulsory psychiatric care of the resident population in their official catchment area.

In the model hospital each patient is assigned to a continuous multi-professional treatment team that is responsible for this patient’s treatment over time, e.g., a new treatment contact after a year, and across all treatment sectors, i.e., inpatient, outpatient, and day care. Hereby a higher relational continuity shall be ensured to a patient within a sector, e.g., if a change of wards occurs, and when a patient changes between treatment sectors, e.g., if a patient stays in day-care or contacts the outpatient service.

The control hospital does not provide this structural therapeutic personnel feature. The staff is usually assigned to a defined therapeutic working setting in one sector, e.g., inpatient ward or outpatient clinic. But a patient can experience an ‘uncoordinated’ relational continuity in the control clinic due to staff rotation or substitution as well.

Patients treated in the psychiatric hospital of their catchment area usually continue their treatment with this hospital, either as inpatient, outpatient, or in day care, and outside office hours in emergency cases. But they can seek treatment in a hospital outside their catchment area or visit psychiatrists in their private practices.

### Inclusion criteria

Eligible for participation in the cohort study were all newly admitted inpatients, 18 years or older, during the recruitment period with a psychiatric diagnosis as defined by ICD-10 (F0 – F7) regardless of their health insurance.

### Exclusion criteria

Not eligible for participation in the cohort study were patients without written consent to participation and data collection, patients not capable of being surveyed, a hospital stay of less than 2 days, a residence outside the hospital’s catchment area, or lack of a permanent residence.

### Recruitment

Recruitment lasted 6 months in both hospitals. During recruitment 1235 patients meeting inclusion criteria were admitted to the hospitals, but 228 (18.5%) of these patients met exclusion criteria. The remaining 1007 patients got invited to participate in the cohort study. 435 patients were able and agreed to participate in the cohort study. 323 of the participating patients of the cohort study had at least one following treatment contact after recruitment with the hospital and the continuity of care measure could be calculated. These 323 study patients were analyzed to answer the research question we address here.

All 435 participating patients of the cohort study were invited after 10 months, and after 20 months for a follow up interview conducted in the hospital. If a participating patient was hospitalized as inpatient or for day care at the time for follow-up the interview was offered and conducted in the hospital, all other participating patients were invited by letter and or telephone to schedule the follow-up with the psychiatrist or psychiatric resident of their treatment team at recruitment. The follow-up interview was completed (patient-rated questionnaires and basic documentation) by the study assistant.

### Survey instruments

#### Patient data

Data on sociodemographic characteristics, diagnoses, and medical history were asked from the patient and completed from the patient clinical records.

The psychiatric diagnoses were diagnosed using the diagnostic criteria for research of the ICD-10 Classification of Mental and Behavioural Disorders [35] by trained psychiatrists as well as psychiatric residents and supervised by senior psychiatrists. Assignment to the main psychiatric diagnosis group was based on the dominant diagnosis at the time of recruitment according to the ICD-10 diagnostic criteria.

#### Continuity of care measure

The assessment of the continuity of care is based on the COC (continuity of care)-index according to Bice and Boxerman [36] who provided an operational definition of continuity of care for a quantitative measure. We adapted their concept to the specifics of the context and data availability of the study hospitals. Our continuity of care measure was defined, surveyed and calculated identically for all study patients. Treatment continuity was ensured in our operationalization if a treatment was carried out again by the responsible senior psychiatrist from the initial treatment team of the ‘continuous multi-professional treatment team’ in the model hospital or the treatment team in the respective setting in the control hospital. The initially responsible senior psychiatrist was identified by the patient’s discharge letter from the inpatient stay during recruitment. The responsible senior psychiatrist was identified in the same way for any further contact of the patient with the hospital by the patient’s following discharge letters from inpatient or day care stays or outpatient reports from outpatient contacts. Discharge letters and outpatient reports are written by the attending psychiatrist or psychiatric resident of the treatment team for which the senior psychiatrist is responsible. The senior psychiatrist corrects and signs discharge letters and outpatient reports which are integrated in the patient clinical records. All contacts were covered by discharge letters or outpatient reports.

The degree of care continuity was calculated dividing the number of all treatment contacts as inpatient, outpatient, or during day care with the initially responsible senior psychiatrist by the total number of all treatment contacts as inpatient, outpatient, or during day care of a patient in the hospital during the observation period. The resulting variable ‘continuity of care’ is a metric variable with values between 0 and 1 with 0 indicating no continuity of care in the contacts and 1 indicating all contacts were with the same treatment team. Outpatient contacts were only included if they took place during regular office hours from Monday through Friday between 8 and 17 o’clock. Emergency outpatient contacts outside of office hours were provided by the hospitals, but these contacts were not included in the calculation of the continuity of care measure because neither the hospital nor a patient could achieve continuity of care due to daily changes in the psychiatrists in charge.

### Outcome measures

Symptom severity was documented using the observer-rated Clinical Global Impression (CGI) rating scales [37] with possible scores ranging from 0–7 and with higher scores indicating higher symptom severity, functioning was documented using the observer-rated Global Assessment of Functioning (GAF) scale [38] with possible scores ranging from 1–100 and with higher scores indicating higher functioning, and health related quality of life was documented using the patient-rated Euro Quality of Life (EQ-VAS) [39] with possible scores ranging from 0–100 and with higher scores indicating higher quality of life. The outcome measures were collected at all three points in time. The observer-rated CGI and GAF scales were scored by the treating psychiatrist or psychiatric resident and the self-rated EQ-VAS was rated by the patient. The CGI as well as the GAF are part of the basic diagnostic procedures in both hospitals and psychiatrists and psychiatric residents are trained in rating.

### Analyses

To answer our research question on continuity of care and clinical outcome measures we analyzed all those participating patients of our cohort study with two or more treatment contacts in the observation period, hereinafter referred to as ‘study patients’. For analyses, the variable continuity of care was median-dichotomized to distinguish the study patients irrespective of their hospital status into two patient groups, patients with a higher degree of continuity of care and patients with a lower degree.

First, we describe the study patients by reporting mean and standard deviation respectively numbers and percentages, separately for the group of study patients with a higher degree of continuity of care and the group of study patients with a lower degree of continuity of care.

To investigate group differences between study patients with a higher and lower degree of continuity of care, the Mann–Whitney-U-test was used for interval-scaled data and the Chi^2^-test for nominal-scaled data. Significance level was set at 0.05.

We describe the differences between both groups for all study patients and separately for only those study patients who could be included with data for all three survey time points and the independent variables in at least one of the three analyses of variance for the outcome measures. An imputation procedure was not applied.

Finally, three analyses of variance with repeated measurements (three points in time) were developed for the association between the patient- (EQ-VAS) or observer-rated (CGI, GAF) outcome measures and continuity of care (median dichotomized) as between-subject factor controlling for the independent variables age (18 to 40 years, 41 to 55 years, older than 55 years), sex (female, male), migration background (with, without), main treatment diagnosis (psychoactive substance use (ICD-10 F1), general psychiatric disorders (ICD-10 F2-7)), duration of psychiatric disease (less than 1 year, 1 to 10 years, more than 10 years), and hospital (model hospital, control hospital).

All analyses were performed using IBM SPSS versions 26.

## Results

### Continuity of care measure and study patients

Out of 435 participating patients of the cohort study, 323 study patients (74.3%) had two or more treatment contacts in the observation period and could be included in the continuity of care analyses. The continuity of care for all 323 study patients ranges from 0.0 to 1.0 with a mean of 0.55 and a median of 0.67 (Table 1). The median-dichotomization resulted in a group of 158 study patients with a higher continuity of care ranging from 0.1 to 1.0 and a mean of 0.95 and a median of 1.0. The group with a lower continuity of care includes 165 study patients with continuity of care ranging from 0.0 to 0.67 and a mean of 0.17 and a median of 0.0.

**Table 1:** Continuity of care and number of contacts of the study patients and both subgroups during the 20 months observation period

		**Total**	**Patient group with higher continuity of care**	**Patient group with lower continuity of care**	***p*** **-value**
**Patients**	**n**	323	158	165	
** Continuity of care**	**mean (SD** ^a^ **)**	0.55 (0.425)	0.95 (0.082)	0.17 (0.213)	< 0.001
	**median**	0.67	1.0	0.0	
	**range**	0.0–1.0	0.71–1.0	0.0–0.67	
** Number of inpatient stays (initial stay included)**	**mean (SD)**	3.01 (3.062)	2.55 (2.550)	3.44 (3.435)	0.009
	**median**	2	2	2	
	**range**	1–22	1–19	1–22	
** Days of inpatient treatment (initial stay included)**	**mean (SD)**	78.01 (74.831)	73.56 (76.122)	82.27 (73.553)	0.296
	**median**	51.00	44.50	61.00	
	**range**	4–417	4–417	6–397	
** Number of day care stays**	**mean (SD)**	0.69 (1.059)	0.84 (1.038)	0.55 (1.062)	0.012
	**median**	0.00	1	0	
	**range**	0–8	0–6	0–8	
** Number of outpatient contacts**	**mean (SD)**	7.50 (12.448)	9.85 (14.657)	5.24 (9.384)	< 0.001
	**median**	3	4	1	
	**range**	0–91	0–91	0–65	

^a^ *SD* standard deviation

Not all 323 study patients could be included in the three analyses of variance due to lack of follow-up participation as well as missing data for the specific data required for analysis.

240 of 323 study patients participated in the first follow-up and 203 in the second follow-up. The data availability for the three outcome measures are given in Table 2.

**Table 2:** Number of study patients participating in the follow-ups and data availability for the three outcome measures

	**Study patients ‘ participation**	**Data available CGI**	**Data available GAF**	**Data available EQ-VAS**
t0 recruitment	323	306	307	309
t1 first follow-up after 10 months	240	181	182	212
t2 second follow-up after 20 months	203	98	97	173

Table 3 shows the continuity of care of those 162 study patients who could be included in at least one of the three analyses of variance of the outcome measures. Their numbers of inpatient stays (3.48) as well as day care stays (0.82) and outpatient contacts (9.68) were higher than among all 323 study patients with 3.01, 0.69, and 7.50 respectively. The group differences between patients with higher compared to lower continuity of care were significantly different in number of contacts in all three care settings, inpatient, day care stay, and outpatient contacts. This applied to both all study patients and the 162 study patients included in the analyses of variance.

**Table 3:** Continuity of care and number of contacts during the 20 months observation period of study patients with at least one outcome measure included in an analysis of variance

		**Total**	**Patient group with higher continuity of care**	**Patient group with lower continuity of care**	***p*** **-value**
**Patients**	**n**	162	78	84	
** Continuity of care**	**mean (SD** ^a^ **)**	0.55 (0.422)	0.95 (0.083)	0.17 (0.210)	< 0.001
	**median**	0.64	1	0.10	
	**range**	0.0–1.0	0.71–1.00	0.0–0.67	
** Number of inpatient stays (initial stay included)**	**mean (SD)**	3.48 (3.772)	2.69 (2.880)	4.21 (4.333)	0.010
	**median**	2	2	3	
	**range**	1–22	1–19	1–22	
** Days of inpatient treatment (initial stay included)**	**mean (SD)**	89,01 (84.652)	84.94 (86.340)	92.80 (83.392)	0.556
	**median**	62.50	50.50	66.00	
	**range**	4–417	4–417	11–397	
** Number of day care stays**	**mean (SD)**	0.82 (1.256)	1.00 (1.227)	0.65 (1.266)	0.080
	**median**	0	1	0	
	**range**	0–8	0–6	0–8	
** Number of outpatient contacts**	**mean (SD)**	9.68 (14.493))	12.69 (16.466)	6.88 (11.810)	0.010
	**median**	5	8	3	
	**range**	0–91	0–91	0–65	

^a^ *SD* standard deviation

Sociodemographic characteristics (Table 4) were distributed equally between both continuity groups with a mean age of 45.2 years, 45.5 female patients and 34.3% with a migration background. The duration of the psychiatric disease history with 22.0% shorter than 1 year and 38.9% longer than 10 years was equally distributed as well. As expected, significantly more patients in the model hospital (87.3%) received a higher continuity of care than patients in the control hospital (12.7%) (*p* < 0.001). In the higher continuity of care group are significantly more patients (43.0%) with mental and behavioral disorders due to psychoactive substance use (ICD-10 F1) and less patients (56.3%) with general psychiatric disorders (ICD-10 F2-F7) than in the lower continuity of care group with 23.6% and 74.5% respectively. Only 4 patients with organic, including symptomatic, mental disorders (ICD-10 F0) were among the 323 study patients. During the 20 months observation period, 16.7% of the 323 study patients changed the dominant diagnosis with a change of the main diagnosis group (substance use and general psychiatric disorders). But for analysis they remained in the assigned main diagnostic group according to the main diagnosis at recruitment.

**Table 4:** Characteristics of all study patients at baseline with two or more treatment contacts in the observation period

	**Total**	**Higher continuity of care**	**Lower continuity of care**	***p*** **-value**
**Patients**, n	323	158	165	
**Age** in years, mean (SD^a^)	45.2 (15.4)	44.6 (14.0)	45.9 (16.7)	0.579
**Sex**, n (%)				
female	147 (45.5)	69 (43.7)	78 (47.3)	0.516
male	176 (54.5)	89 (56.3)	87 (52.7)	
**Migration background**, n (%)				
yes	110 (34.3)	61 (38.6	49 (30.1)	0.107
no	211 (65.7)	97 (61.4)	114 (69.9)	
**Main treatment diagnosis,** n (%)				
Organic, including symptomatic, mental disorders (ICD-10 F0)	4 (1.2)	1 (0.6)	3 (1.8)	0.001
mental and behavioral disorders due to psychoactive substance use (ICD-10 F1)	107 (33.1)	68 (43.0)	39 (23.6)	
general psychiatric disorders (ICD-10 F2-7)	212 (65.6)	89 (56.3)	123 (74.5)	
thereof:	–––	–––	–––	–––
F2 schizophrenia, schizotypal and delusional disorders	61 (28.8)	26 (29.2)	35 (28.5)	
F3 mood (affective) disorders	96 (45.3)	40 (44.9)	56 (45.5)	
F4 neurotic, stress-related and somatoform disorders	20 (9.4)	12 (13.5)	8 (6.5)	
F6 disorders of adult personality and behavior	35 (16.5)	11 (12.4)	24 (19.5)	
**Duration of psychiatric disease**, n (%)				
< 1 year	69 (22.0)	34 (22.1)	35 (21.9)	0.708
1–10 years	123 (39.2)	57 (37.0)	66 (43.3)	
> 10 years	122 (38.9)	63 (40.9)	59 (36.9)	
**Hospital**, n (%)				
model hospital	183 (56.7)	138 (87.3)	45 (27.3)	< 0.001
control hospital	140 (43.3)	20 (12.7)	120 (72.7)	

^a^ *SD* standard deviation

Table 5 shows the sociodemographic characteristics and duration of psychiatric disorders and the diagnostic group distribution for the 162 study patients included in an analysis of variance. They are older with a mean age of 46.8 years, more female patients (53.1%) and 35.2 have a migration background. The main diagnostic groups are more even distributed with 32.1% of patients with mental and behavioral disorders due to psychoactive substance use (ICD-10 F1) and 67.9% with general psychiatric disorders (ICD-10 F2-F7) in the higher continuity of care group compared to 20.2% and 78.6% respectively in the lower continuity of care group.

**Table 5:** Characteristics of the study patients at baseline with two or more treatment contacts in the observation period included in an analysis of variance

	**Total**	**Higher continuity of care**	**Lower continuity of care**	***p*** **-value**
**Patients**, n	162	78	84	
**Age** in years, mean (SD^a^)	46.8 (13.7)	46.0 (13.4)	47.6 (14.1)	0.465
**Sex**, n (%)				
female	86 (53.1)	42 (53.8)	44 (52.4)	0.876
male	76 (46.9)	36 (46.2)	40 (47.6)	
**Migration background**, n (%)				
yes	57 (35.2)	29 (37.2)	28 (33.7)	0.742
no	104 (64.2)	49 (62.8)	55 (66.3)	
**Main treatment diagnosis,** n (%)				
Organic, including symptomatic, mental disorders (ICD-10 F0)	1 (0.6)	0	1 (1.2)	0.155
mental and behavioral disorders due to psychoactive substance use (ICD-10 F1)	42 (25.9)	25 (32.1)	17 (20.2)	
general psychiatric disorders (ICD-10 F2-7)	119 (74.5)	53 (67.9)	66 (78.6)	
thereof:	–––	–––	–––	–––
F2 schizophrenia, schizotypal and delusional disorders	39 (32.8)	18 (34.0)	21 (31.8)	
F3 mood (affective) disorders	52 (43.7)	22 (41.5)	30 (45.5)	
F4 neurotic, stress-related and somatoform disorders	12 (10.1)	7 (13.2)	5 (7.6)	
F6 disorders of adult personality and behavior	16 (13.4)	6 (11.3)	10 (15.2)	
**Duration of psychiatric disease**, n (%)				
< 1 year	32 (19.8)	17 (22.4)	15 (18.3)	0.809
1–10 years	59 (36.4)	28 (36.8)	31 (37.8)	
> 10 years	67 (41.4)	31 (40.8)	36 (43.9)	
**Hospital**, n (%)				
model hospital	93 (57.4)	68 (87.2)	25 (29.8)	< 0.001
control hospital	69 (42.6)	10 (12.8)	59 (70.2)	

^a^ *SD* standard deviation

16 study patients (5.0%) reported contact with a psychiatric hospital outside their catchment area and 25 (7.7%) reported a visit of a psychiatrist in private practice. These contacts are not included in the calculation of the continuity of care measure. No patient was treated at both hospitals in the study.

### Outcome measures

Table 6 shows the scores of the three outcome measures for all three time points of those study patients with their data being included in the respective analysis of variance with repeated measurements. The analysis of variance could be performed with data of 78 study patients for the outcome measure CGI, of 76 study patients for the GAF, and of 136 study patients for the EQ-VAS.

**Table 6:** Outcome measure values for CGI, GAF, and EQ-VAS in the observation period

		**Total**		**Higher continuity**		**Lower continuity**	
		Mean	SD^a^	Mean	SD	Mean	SD
**Symptom severity CGI**^**b**^	*n*	78		48		30	
	t0	5.1	0.83	5.1	0.90	5.0	0.72
	t1 after 10 months	4.4	1.35	4.2	1.47	4.7	1.09
	t2 after 20 months	4.5	1.11	4.4	1.14	4.8	1.03
**Level of functioning GAF**^**c**^	*N*	76		46		30	
	t0	44.8	14.80	44.1	14.47	45.8	15.48
	t1 after 10 months	55.6	16.42	59.3	16.65	50.0	14.61
	t2 after 20 months	55.6	16.23	59.1	14.38	50.2	17.61
**Quality of life EQ-VAS**^**d**^	*N*	136		62		74	
	t0	52.8	25.16	55.9	24.70	50.1	25.40
	t1 after 10 months	63.4	23.03	64.7	22.45	62.2	23.58
	t2 after 20 months	64.7	22.57	66.0	22.04	63.7	23.10

^a^ *SD* standard deviation

^b^ observer-rated; possible CGI scores range from 0–7, with higher scores indicating higher symptom severity

^c^ observer-rated; possible GAF scores range from 1–100, with higher scores indicating higher functioning

^d^ patient-rated; possible EQ-VAS scores range from 0–100, with higher scores indicating higher quality of life

Symptom severity (CGI) was even in both groups at recruitment (5.1 and 5.0) and decreased over time to 4.4 in higher continuity group and to 4.8 in the group with lower continuity. The level of functioning (GAF) showed a slightly lower score (44.1) in the higher continuity group at recruitment than in the lower continuity group (45.8). Over time the higher continuity group rose to a higher functioning scoring (59.1) while the lower continuity group gained only 4.4 points scoring 50.2 at the end of the observation period. The patient-rated quality of life (EQ-VAS) gained 10.1 points to a score of 66.0 in the higher continuity group while in the lower continuity group it rose by 13.6 to a score of 63.7.

The analyses of variance with repeated measurements showed a significant within subject effect of time on symptom severity (CGI: df 2, F 5.060, *p* 0.008) and quality of life (EQ-VAS: Greenhouse–Geisser corrected df 1.821, F 15.930, *p* < 0.001) but none on the level of functioning (GAF: df 2, F 2.040, *p* 0.134), and none of the three outcome measures showed a significant within subject effect of time with continuity of care.

Table 7 presents the main results of the effects of continuity of care as between-subject factor on the respective outcome measures as well as the values of all further six factors integrated in the model.

**Table 7:** Results of the analyses of variance with repeated measurements of continuity of care as between-subject factor on the outcome measures CGI, GAF, and EQ-VAS

		**Type III Sum of Squares**	**df**	**Mean Square**	**F**	**Sig**	**Partial Eta**^**2**^
**Symptom severity: CGI (** ***n*** ** = 78)**	**Continuity of care**	**8.775**	**1**	**8.775**	**4.565**	**0.036**	**0.064**
	Age	6.402	2	3.201	1.665	0.197	0.047
	Sex	0.796	1	0.796	0.414	0.522	0.006
	Migration background	7.469	1	7.469	3.886	0.053	0.055
	Main treatment diagnosis	0.634	1	0.634	0.330	0.568	0.005
	Duration of psychiatric disease	22.028	2	11.014	5.730	0.005	0.146
	Hospital	6.677	1	6.677	3.474	0.067	0.049
	Constant value	1581.161	1	1581.161	822.548	0.000	0.925
	Error	128.792	67	1.922			
**Level of functioning: GAF (** ***n*** ** = 76)**	**Continuity of care**	**2131.751**	**1**	**2131.751**	**5.453**	**0.023**	**0.076**
	Age	2401.894	2	1200.947	3.072	0.053	0.085
	Sex	183.995	1	183.995	0.471	0.495	0.007
	Migration background	72.513	1	72.513	0.185	0.668	0.003
	Main treatment diagnosis	841.362	1	841.362	2.152	0.147	0.032
	Duration of psychiatric disease	2702.041	2	1351.020	3.456	0.037	0.095
	Hospital	1479.457	1	1479.457	3.784	0.056	0.054
	Constant value	232584.845	1	232584.845	594.910	0.000	0.900
	Error	25803.222	66	390.958			
**Quality of life EQ-VAS: (** ***n*** ** = 136)**	**Continuity of care**	**3127.831**	**1**	**3127.831**	**2.875**	**0.092**	**0.022**
	Age	1353.724	2	676.862	0.622	0.538	0.010
	Sex	1411.622	1	1411.622	1.298	0.257	0.010
	Migration background	6371.384	1	6371.384	5.856	0.017	0.044
	Main treatment diagnosis	652.071	1	652.071	0.599	0.440	0.005
	Duration of psychiatric disease	3374.960	2	1687.480	1.551	0.216	0.024
	Hospital	4316.274	1	4316.274	3.967	0.049	0.031
	Constant value	937308.776	1	937308.776	861.534	0.000	0.872
	Error	137082.182	126	1087.954			

Symptom severity (CGI) differed significantly (*p* 0.036) with a medium effect size (partial Eta^2^ 0.064) between both continuity groups with a larger effect in the higher continuity group. The level of functioning (GAF) differed as well significantly (*p* 0.023) with a medium effect size (partial Eta^2^ 0.076) between both continuity groups with a larger effect in the higher continuity group. Quality of life (EQ-VAS) did not reach a significant difference (*p* 0.092) with only a small effect size (partial Eta^2^ 0.022).

The duration of psychiatric disease effected as well significantly on CGI (p 0.005) and GAF (*p* 0.037), both scoring with clinically better values in the higher continuity group than the lower one, but the longer the psychiatric diseases lasted the more severe were symptoms and the lower was the level of functioning. Quality of life rose higher during the observation period in the group with higher continuity compared to the lower continuity group, but a migration history effected significantly (*p* 0.017) the rise of quality-of-life values with lower levels in patients with that background compared to those without. The hospital showed a significant (*p* 0.049) effect on quality of life with the model hospital rising on a lower level than did the control hospital. Due to the small number of study patients with complete data for the analyses of variance with repeated measurements, the statistical power with alpha set on 0.5 was 0.5 for the CGI, 0.6 for the GAF, and 0.3 for the EQ-VAS.

## Discussion

Relational continuity of care affected in our cohort study the patients’ symptom severity, social functioning, and quality of life. A higher relational continuity of care reduced the symptom severity and enhanced the social functioning with a medium effect size significantly more than in patients experiencing a lower continuity. We could show a positive effect on quality of life as well but with only a small effect size not reaching statistical significance. These findings go along with previous findings in the international literature and study results from Germany with corresponding care services [8, 9, 11, 12, 22, 23, 26].

The analyses of variance showed that time itself had a significant within-subject effect in a clinically favorable direction for symptom severity and quality of life, not though for social functioning. But the interaction between time and continuity of care had no significant effect on the outcome measures, suggesting a time-independent effect of continuity of care.

Apart from continuity of care, only the duration of psychiatric disease, as one of the six factors controlled for in the analyses of variance, showed a favorable significant and medium effect. It got along with reduced symptom severity and higher social functioning. But this effect decreased with increasing duration of the psychiatric disease confirming clinical experience with chronic courses. In contrast, an existing migration background affected significantly unfavorably quality of life suggesting a higher vulnerability of this group.

Our methodological approach defining two continuity groups of patients by median-dichotomization resulted in two groups with contrastively different intensities of continuity of care. The group with a higher level reached a 95% degree of continuity of care with the initially responsible senior psychiatrist while the group with lower continuity only reached 17%.

Comparing the groups’ sociodemographic and clinical characteristics, and their number of contacts in the treatment sectors might indicate patient characteristics and health care service structures favoring a higher degree of relational continuity of care. The sociodemographic factors age, sex, and migration background did not differ between both groups, nor did the duration of the psychiatric disease. It might suggest that these patient characteristics per se do not imply a higher or lower degree of continuity, and that the mental health service and its professionals do no select unequally patients in this respect. But patients with higher continuity of care were more often in contact due to more outpatient contacts and relatively fewer inpatient stays. Those with a lower continuity tended to be more often inpatient with longer stays and considerably less in outpatient contact. Furthermore, the treating hospital, and the diagnosis group made a difference pointing out to specifically arranged psychiatric care services. The model hospital takes long psychiatric disease durations with recurrent treatment episodes into account. It actively arranges treatment teams to enhance relational continuity of care by working with patients across treatment sectors and it fosters day care stays and especially outpatient contacts, confirming the respective results of the studies on this service structure [30–34]. The higher proportion of patients with mental and behavioral disorders due to psychoactive substance use in the higher continuity group might surprise. But psychiatric hospitals, irrespective of model hospital status, often organize care services with wards and teams specifically for this patient group in order to cope with clinical symptoms and their interaction behavior, enhancing hereby the continuity of care. These results indicate that actively implemented health care services with specific features can contribute to rising continuity of care for patients.

### Strengths and weaknesses of the study

The study’s strength is its design as a cohort study in the realm of health care services research with a broad approach on psychiatric patients as served by the mental health services in Germany. Its limitation is the low statistical power for the analyses of variance with repeated measures due to a small sample size as a result of non-participation. The overall number of study patients with data for all three points in time does not permit in-depth statistical analyses of diagnostic subgroups such as ICD-10 F2 schizophrenia or F3 mood (affective) disorders in the analyses of variance although relevant to continuity of care [31, 40].

Non-participation is a result of exclusion criteria, the patients’ ability and willingness to participate in the study and attending 2 follow-up contacts providing outcome data for all three points in time, necessary for analyses. These aspects affect inherently the research subject ‘relational continuity’, excluding more likely patients with lower relational continuity of care combined with more severe symptoms, unstable social relations and living environment. The resulting bias probably diminished differences between patients with higher and lower continuity of care in our study. Considering this bias, a methodological approach with more information on non-participants’ sociodemographic and clinical characteristics, and contact-frequencies is advisable but has to respect ethical aspects of data protection.

Furthermore, patients with organic, including symptomatic, mental disorders (ICD-10 F0) were excluded entirely due to non-participation. Further studies on continuity of care in this patient group are necessary with a specifically adapted conduct of the study with an active involvement of caregivers and curators, modified survey instruments, and flexible follow-up interview settings.

## Conclusions

Our study results support continuity of care as a favorable clinical aspect in psychiatric patient treatment and encourage mental health care services to consider health service delivery structures that increase continuity of care in the psychiatric patient treatment course. Patients’ motives as well as methodological reasons for non-participation remain considerable sources for bias in psychiatric health care services research.

## Data Availability

The datasets used and/or analyzed during the current study are available from the corresponding author on reasonable request.

## Abbreviations

ACT: Assertive Community Treatment
CGI: Clinical Global Impression Rating Scales
COC: Continuity of Care
EQ-VAS: Euro Quality of Life Visual Analogue Scale
GAF: Global Assessment of Functioning
ICD-10: International Statistical Classification of Diseases and Related Health Problems, 10th revision
